# Cardiovascular Imaging-Derived Skeletal Muscle Mass Correlates With Fitness and Survival in Patients With Univentricular Circulation

**DOI:** 10.7759/cureus.56276

**Published:** 2024-03-16

**Authors:** Vojtěch Illinger, Kryštof Slabý, Vojtěch Suchánek, Jiří Radvanský

**Affiliations:** 1 Department of Rehabilitation and Sports Medicine, Second Faculty of Medicine, Charles University, Motol University Hospital, Prague, CZE; 2 Department of Imaging Methods, Second Faculty of Medicine, Charles University, Motol University Hospital, Prague, CZE

**Keywords:** exercise intolerance, mortality, skeletal cross-sectional area, thoracic muscle mass, total cavopulmonary connection, sarcopenia, magnetic resonance imaging, computed tomography

## Abstract

Aims

This study aims to retrospectively quantify skeletal muscle mass from cardiovascular imaging studies in total cavopulmonary connection (TCPC) patients and to correlate calculated muscle mass with clinical outcomes.

Materials and methods

Ninety-one TCPC patients at a mean age of 24.0 ±5.5 years (37 women; 40.7%) who underwent chest computed tomography (CT) or cardiac magnetic resonance imaging (MRI) as part of their follow-up were identified in a single-center database. The cross-sectional skeletal muscle index (SMI) at the Th4 and Th12 levels was calculated from CT images, and the dorsal skeletal muscle area (SMA) at the Th12 level was measured from an MRI.

Results

Calculated SMI at Th12 level was 38.0 (34.5; 42.0) cm^2^.m^-2^ or 89.6 (81.9; 101.6) % of predicted values. The median follow-up from CT was 5.9 (3.1; 8.5) years, and the composite endpoint (death N=5, heart transplant N=6) was reached in a total of 11 (26.8%) patients. Patients with SMI (Th12) less than 90% of predicted values had a hazard ratio of 5.8 (95% CI: 1.2; 28.3) (p=0.03) for endpoint achievement. In the MRI group, dorsal SMA at the Th12 level was 27.6 ±5.1 cm^2^ in men and 20.0 ±5.8 cm^2^ in women. Correlations were found between SMA/kg and peak oxygen uptake (VO_2 _peak) (r=0.48, p=0.0005) and fat-free mass (r=0.63, p<0.0001), respectively.

Conclusions

A low SMI at the Th12 level was associated with a higher risk of death or cardiac transplantation. Evaluation of skeletal muscle mass using cardiovascular imaging methods allows rapid identification of individuals at risk of sarcopenia.

## Introduction

Low exercise tolerance and sarcopenia are major factors contributing to morbidity and quality of life in patients with total cavopulmonary connection (TCPC) [1-3]. Many macroscopic and metabolic changes in skeletal muscle are observed in patients with univentricular circulation. Muscle mass is reduced in TCPC patients; 13 out of 16 adult patients with TCPC in Cordina's study had reduced skeletal muscle mass on whole-body densitometry, four (25%) of whom met the diagnostic criteria for sarcopenia [4]. Patients with Fontan circulation have lower muscle strength on the handgrip test compared to healthy controls, increased skeletal muscle sympathetic activity, increased systemic vascular resistance, higher serum norepinephrine levels, and decreased vascular conductance [5,6]. Muscle cell changes have not yet been demonstrated immunohistochemically in patients with TCPC, but a similar mechanism of metabolic alteration and conversion of fast twitch oxidative muscle fibers (type IIa) to fast glycolytic (type IIx) as described by Middlekauff in patients with "conventional" chronic heart failure can be assumed [7]. Impaired post-exercise phosphocreatine resynthesis on phosphorus ^31^P spectroscopy in TCPC patients versus healthy controls is also indicative of altered muscle metabolism [4]. Compared to healthy subjects, TCPC patients not only have lower resting muscle hemoglobin saturation measured by infrared spectroscopy but also slower muscle desaturation kinetics during exercise, implying an alteration of muscle metabolism and worse oxygen utilization in working muscle [8].

Several studies have demonstrated the feasibility and reliability of skeletal muscle measurements from both computed tomography (CT) and magnetic resonance imaging (MRI), and normative data are available for standardized slices [9-11]. Evaluation of skeletal muscle mass from cardiovascular imaging routinely performed during TCPC patient follow-up would allow rapid identification of individuals at risk of sarcopenia.

## Materials and methods

In the single-center nationwide database of TCPC patients, 91 patients (37 women; 40.7%) underwent stage III of single-chamber palliation by TCPC and had a CT chest examination (CT group) or cardiac MRI (MRI group) as part of their follow-up.

The CT group comprised 41 TCPC patients (15 women; 36.5%); the median age at the CT was 19.7 (17.4; 23.3) years. Cardiopulmonary exercise test results ±2 years within the CT study date were included in the analysis. In 20 (48.8%) patients, TCPC was completed using an extracardiac conduit; in the remaining 21 patients, an intracardiac tunnel was used; and in 15 (36.5%) patients, fenestration was created at the surgery. The median age at TCPC completion was 4.59 (3.3; 6.5) years. The total number of sternotomies was 3.0 (2.0; 4.0). The representation of diagnoses in the cohort is listed in Table 1. The mean height of the patients was 169.7 ± 11.1 cm, their weight was 64.3 ± 16.3 kg, and their body mass index was 22.2 ± 2.6 kg.m^-2^.

**Table 1:** Patient diagnoses in the CT group CT: computed tomography

Congenital heart defect	Number of patients	Percent
Double-outlet right ventricle	9	22.0
Tricuspid atresia	9	22.0
Double-inlet left ventricle	8	19.5
Hypoplastic left heart syndrome	5	12.2
Complete atrioventricular septal defect	3	7.3
Congenitally corrected transposition of the great arteries	2	4.9
Double-inlet right ventricle	1	2.4
Mitral atresia	1	2.4
Pulmonary atresia with intact interventricular septum	1	2.4
Hypoplastic right ventricle	1	2.4
Transposition of the great arteries	1	2.4
Total	41	100.0

The MRI group consisted of 50 TCPC patients (22 women; 44.0%) with surgery dates between 1993 and 2003 who underwent cardiopulmonary exercise testing (CPET), bioimpedance analysis of body composition, echocardiography, and cardiac MRI during their follow-up visit at a tertiary heart center.

The mean age of the patients at examination was 26.3 ± 4.4 years. Body mass index at examination was 23.4 ± 4.2 kg.m^-2^. In 46 patients (92.0%), TCPC was performed using an extracardiac conduit, and in the remaining four patients, using an intracardiac tunnel. Nineteen patients (38.0%) had a morphologically right systemic ventricle, and the median number of sternotomies was 3.0 (2.0; 4.0).

CPET

Exercise stress testing on an electromechanically braked bicycle ergometer (Ergoline Ergoselect 150 or 200, Bitz, Germany) with analysis of ventilation and exhaled O_2_ and CO_2_ concentration by the "breath-by-breath" method (Oxycon Pro with an electrochemical oxygen sensor, Jaeger, Germany) was performed until patient exhaustion using an incremental protocol of +0.5 W/kg of body weight every three minutes. Criteria for maximal effort were a respiratory exchange ratio at peak exercise >1.05 and a perceived exertion ≥16 on the Borg scale.

Bioimpedance body composition analysis

After five minutes in the supine position, electrodes were attached, and body impedance measurements (Bodystat QuadScan 4000, BodyStat, British Isles) were performed under standard conditions. Using a software algorithm (Bodystat Windows Software v1.1, BodyStat, British Isles), the absolute and relative fat and fat-free mass were evaluated.

Thoracic skeletal muscle mass measurement using CT

**Figure 1:**
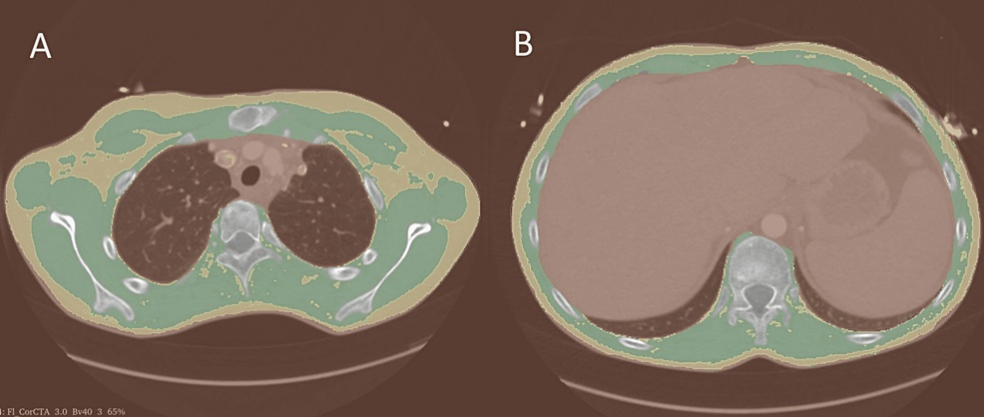
Axial view at the level of the 4th thoracic vertebra (A) and 12th thoracic vertebra (B), segmentation for calculation of SMA (green) and subcutaneous fat mass (ochre), CT SMA: skeletal muscle area, CT: computed tomography

All examinations were performed on a third-generation CT (Somatom Force, Siemens, Germany). In the axial plane, the 4th and 12th thoracic vertebra levels were identified using the 3D Slicer software (3D Slicer v5.2.2). Skeletal muscle and subcutaneous fat were manually segmented, and subsequently, segment area was calculated in the range of -29 to 150 Hounsfield units for muscle and -150 to -30 Hounsfield units for adipose tissue in the Segment Cross-Sectional Area module (3D Slicer v5.2.2) (Figure 1). Only non-contrast protocols were used for muscle and fat tissue segmentation.

Thoracic skeletal muscle mass measurement using cardiac MRI

**Figure 2:**
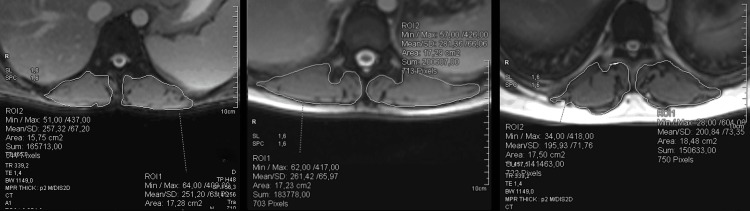
Manual segmentation and region of interest area calculation of the dorsal muscle compartment at the level of the 12th thoracic vertebra (detail, MRI) MRI: magnetic resonance imaging

All examinations were performed on a 1.5 Tesla MRI scanner (Avanto, Siemens, Germany). TRUFI (true fast imaging with steady-state free precession) sequences were used for the identification of the superior mesenteric artery origin and the body of the first lumbar vertebra. Dorsal skeletal musculature (musculi erectores trunci) was then manually segmented in the axial plane, and its area was calculated using the Syngo.via software (Syngo.via VB60, Siemens, Germany) (Figure 2).

Ethical considerations

This retrospective study was conducted in accordance with the principles of the Helsinki Declaration of 1975 and was approved by the Ethics Committee of Motol University Hospital (EK-1166/23). Informed consent was not required due to the retrospective nature of the study with no direct impact on patient management.

Statistical analysis

The test of data distribution was performed using the Shapiro-Wilk test, and the data are appropriately presented as mean ± standard deviation or median (quartile range). Statistica software (Statistica for Windows v12.0, StatSoft Inc., USA) was used for statistical analysis. The relationship between continuous variables was tested by Pearson correlation, and the relationship between categorical and continuous variables was tested by a two-sample t-test, analysis of variance (ANOVA), or Kruskal-Wallis test. The survival analysis was plotted using the Kaplan-Meier method, and the cumulative proportion of survivors in each subgroup was calculated using the Cox regression model. A level of p<0.05 was considered statistically significant.

## Results

Thoracic CT-derived skeletal muscle mass

The skeletal muscle area (SMA) at the Th4 level was 190.4 (152.1; 214.1) cm^2^, and the calculated skeletal muscle index (SMI) was 64.6 (58.5; 70.6) cm^2^.m^-2^. At the Th12 level, SMA was 105.4 (86.7; 121.9) cm^2^ and SMI was 38.0 (34.5; 42.0) cm^2^.m^-2^, corresponding to 89.6 (81.9; 101.6) % of normal, according to Derstine et al. [11].

The median follow-up from CT, echocardiography, and CPET was 5.9 (3.1; 8.5) years, and the composite endpoint (death N=5, heart transplant N=6) was reached in a total of 11 (26.8%) patients, with an age at the endpoint 18.7 (17.1; 25.4) years.

**Figure 3:**
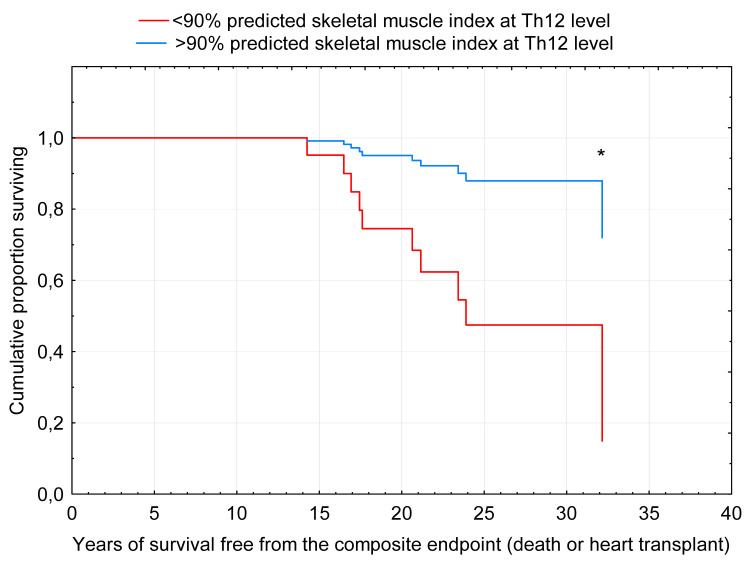
Survival to composite endpoint in patients with >90% predicted SMI Th12 (blue) and <90% predicted SMI Th12 (red), Cox's proportional hazards model SMI: skeletal muscle index, *: p<0.05

Patients with SMI (Th12) less than 90% of predicted values had a hazard ratio (HR) of achieving the composite endpoint of HR=5.8 (95% CI 1.2; 28.3), p=0.03 (Figure 3). There was no correlation between SMI at the Th12 level and the presence of fenestration on examination (p=0.79). No significant relationship was found between SMI at the Th12 level and type of surgery (lateral tunnel vs. extracardiac conduit) (p=0.38), age of TCPC completion (p=0.73), or a number of sternotomies performed (p=0.31).

Peak oxygen consumption during CPET was 24.7 ± 5.6 ml.min^-1^.kg^-1^, translating to 56.4 ± 12.5% of predicted values. At the level of the whole cohort, no significant correlation was found between peak oxygen uptake (VO_2 _peak) and SMI at Th4 or Th12 levels (p=0.71 and p=0.11, respectively).

Cardiac MRI-derived thoracic skeletal muscle mass

The absolute and relative fat-free mass determined by bioimpedance analysis were 61.0 ± 7.9 kg and 84.0 ± 5.7% in men and 44.2 ± 7.3 kg and 71.9 ± 6.4% in women, respectively. Peak oxygen consumption during CPET was 30.7 ± 5.0 ml*min^-1^*kg^-1^ in men and 24.6 ± 6.3 ml*min^-1^*kg^-1^ in women, corresponding to achieving 69.2 ± 14.4% of predicted VO_2_ peak values. The area of the dorsal skeletal muscle segment at the Th12 level on MRI was 27.6 ± 5.1 cm^2^ in men and 20.0 ± 5.8 cm^2^ in women.

**Figure 4:**
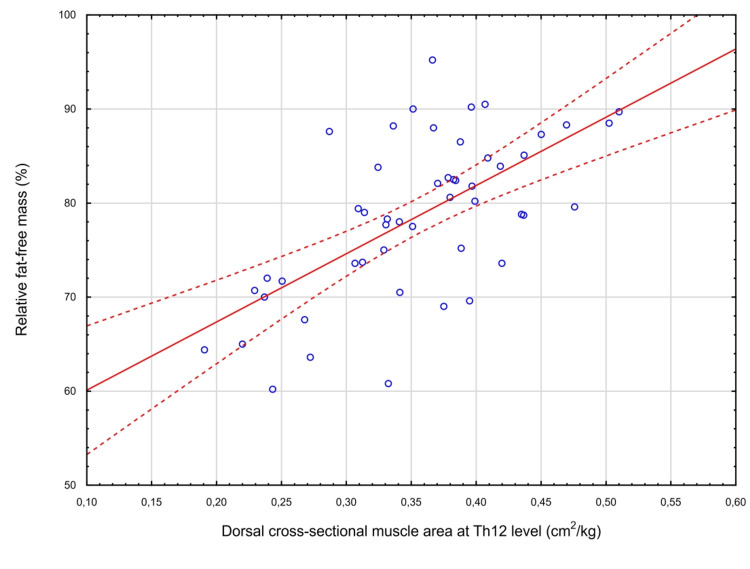
Correlation between the relative amount of fat-free mass (%) measured by whole-body bioimpedance and the area of the dorsal muscle compartment at the level of the 12th thoracic vertebra (cm2 per kg of body weight) at MRI (r=0.63, p<0.0001) MRI: magnetic resonance imaging

There was an inverse correlation between SMA and patient age (r=-0.33, p=0.019). A strong positive correlation was found between the dorsal muscle compartment area indexed for patient body weight and the relative amount of fat-free mass (r=0.63, p<0.0001) (Figure 4), and there was a moderate positive correlation with the VO_2_ peak (ml.min^-1^.kg^-1^) (r=0.48, p=0.0005).

## Discussion

In a retrospective study of 154 patients with Fontan circulation, a lower volume of the large lumbar muscle on an abdominal CT scan was associated with a higher risk of hospitalization for heart failure [12]. Oh et al. found a lower likelihood of postoperative complications and prolonged intensive care bed stays after Fontan surgery in patients with a higher SMI (Th4) detected on preoperative CT scans [13]. However, the method of muscle segmentation was not optimal in this study; only the pectoralis major and spinal extensors were manually segmented. We are not aware of other published data on the use of chest CT to measure muscle mass in TCPC patients.

In our CT cohort, the median SMI (Th12) was below the population norm, according to Derstine et al. (89.6%). However, a surprising finding was that none of the patients in the cohort met the SMI criteria for clinical sarcopenia (cut-off <20.8 cm^2^.m^-2^ and <28.8 cm^2^.m^-2^ in females and males, respectively) [11]. In contrast, when investigated by the reference method (whole-body densitometry), Cordina et al.'s study found a prevalence of sarcopenia of 25% in a small cohort of 16 adult patients with TCPC [4].

The incidence of composite endpoints was relatively high (26.8% of the cohort). This may be due to the selection bias of patients referred for CT scanning outside the standard follow-up protocol. Patients with a cardiac CT scan were also in worse functional status than the national cohort average (56.4% vs. 69.1% predicted VO_2_ peak). The HR for achieving a composite endpoint in patients who had <90% of predicted SMI at the Th12 level was HR=5.8 with a relatively wide confidence interval (1.2; 28.3).

Skeletal muscle mass evaluation should become a routine part of the CT scan description for TCPC patients. Standard body composition testing (bioimpedance or densitometry) may then be indicated if sarcopenia is suspected from the CT. There is emerging evidence for improving exercise tolerance and muscle function via cardiac rehabilitation programs in TCPC patients [14]. However, the amount of thoracic skeletal muscle may not correlate with tissue quality, muscle strength, or the biomechanical efficiency of ventilation.

In a retrospective study of 40 TCPC patients aged 25.5 ± 7.9 years, the SMA of the dorsal muscle compartment (Th12) group was reported to be 33.5 ± 8.4 cm^2^ in men and 25.1 ± 4.9 cm^2^ in women [15]. In our study, we measured lower numbers (27.6 ± 5.1 mm^2^ in men and 20.0 ± 5.8 mm^2^ in women).

Smith et al. reported a positive correlation between MRI-derived skeletal muscle area at (Th4) and VO_2_ peak in TCPC patients [16]. However, their method of segmentation of the pectoralis major and spinal extensors is not adequately described; therefore, the repeatability of the study results is questionable. In our study, the cross-sectional area of the dorsal muscle compartment measured at the Th12 level was moderately positively correlated with the VO_2_ peak.

Given the correlation (r=0.63) between the cross-sectional area of the erectores trunci at the Th12 level and the percentage of fat-free mass measured by whole-body bioimpedance analysis, this simple MRI measurement can be used to estimate fat-free mass in cases where bioimpedance measurements or densitometry are not available.

Future research should be extended to larger cohorts, using emerging advanced volumetric methods and 3D automated tissue segmentation [17].

Limitations

We are aware of the presumed selection bias of patients with CT scans available, as cardiac CT is not a routine part of the standard follow-up protocol. Those patients had lower exercise tolerance and higher mortality than the rest of the national TCPC dataset.

Comparison of our results to those of other studies is difficult due to significant differences in the methodology of measuring skeletal muscle cross-sectional area.

## Conclusions

A low SMI at the Th12 level was associated with a higher risk of death or cardiac transplantation. The area of spinal extensors at the Th12 level correlates with exercise tolerance and fat-free mass. Thoracic CT or MR scan-derived skeletal muscle mass is an easily available sarcopenia screening tool in TCPC patients; thus, it would be advisable to calculate muscle mass routinely as part of every cardiac CT or MR evaluation in these patients. Further research on this topic is warranted, using novel tissue volume segmentation methods.
